# Microstructural Analysis of Novel Preceramic Paper-Derived SiC_f_/SiC Composites

**DOI:** 10.3390/ma14226737

**Published:** 2021-11-09

**Authors:** Ke Li, Egor Kashkarov, Hailiang Ma, Ping Fan, Qiaoli Zhang, Peng Zhang, Jilong Zhang, Zhaohui Wu, Larissa Wahl, Roman Laptev, Andrey Lider, Nahum Travitzky, Daqing Yuan

**Affiliations:** 1 China Institute of Atomic Energy, Beijing 102413, China; libeichen@tpu.ru (K.L.); mhl624@ciae.ac.cn (H.M.); fanping@ciae.ac.cn (P.F.); zql@ciae.ac.cn (Q.Z.); 2 School of Nuclear Science and Engineering, National Research Tomsk Polytechnic University, 634050 Tomsk, Russia; laptev.roman@gmail.com (R.L.); lider@tpu.ru (A.L.); 3 The Institute of High Energy Physics of the Chinese Academy of Sciences, Beijing 100049, China; zhangpeng@ihep.ac.cn; 4 State Nuclear Security Technology Center, Beijing 102401, China; jilongzhang@snstc.org (J.Z.); wuzhaohui521@sina.com (Z.W.); 5 Department of Materials Science, Glass and Ceramics, Friedrich-Alexander-Universität Erlangen-Nürnberg, 91054 Erlangen, Germany; larissa.wahl@fau.de (L.W.); nahum.travitzky@fau.de (N.T.); 6 School of Advanced Manufacturing Technologies, National Research Tomsk Polytechnic University, 634050 Tomsk, Russia

**Keywords:** laminated composite, silicon carbide, microstructures, positron annihilation

## Abstract

This paper presents the results of microstructural analysis of novel preceramic paper-derived SiCf/SiC composites fabricated by spark plasma sintering. The sintering temperature and pressure were 2100/2200 °C and 60/100 MPa, respectively. The content of fibers in the composites was approx. 10 wt %. The SiC_f_/SiC composites were analyzed by positron annihilation methods, X-ray diffraction technology, scanning electron microscopy, and Raman spectroscopy. Longer sintering time causes the proportion of the 6H-SiC composition to increase to ~80%. The increase in sintering temperature from 2100 °C to 2200 °C leads to partial transition of 4H-SiC to 6H-SiC during the sintering process, and the long-life component of positrons indicates the formation of Si vacancies. The Raman characteristic peaks of turbostratic graphite appear in the Raman spectrum of SiC fibers, this is caused by the diffusion of carbon from the surface of the SiC fiber and the preceramic paper during the high-temperature sintering process.

## 1. Introduction

Silicon carbide (SiC) has excellent properties—such as low density, high specific strength, high specific modulus, resistance to thermal shock, low coefficient of thermal expansion, radiation tolerance, and chemical inertness [1,2]. Due to the high radiation resistance at elevated temperature, low activation and decay heat properties, low thermal neutron cross section and low tritium permeability, SiC is of particular interest for applications in nuclear reactors [3]. However, like other ceramic materials, the inherent high brittleness of monolithic SiC has become a disadvantage of its application as a structural material [4].

Reinforcement of SiC matrix composites with continuous SiC fibers leads to the quasi-ductile behavior of the material under mechanical loading and the reduction of the macroscopic brittleness of the composite. Therefore, the SiC_f_/SiC composites with more advantageous strength reliability and damage tolerance properties are being developed for nuclear energy and aerospace fields [5]. The common approach is to fabricate the SiC-based composite materials consisting of the continuous or dispersed (short) fiber phase, the continuous matrix phase, and interface layer between the matrix and the fiber [6]. The implementation of so-called interface layer coating—such as pyrolytic carbon or boron nitride deposited on the surface of the SiC fibers (SiC_f_)—can improve the macroscopic mechanical properties of the SiC_f_/SiC composites by preventing the integration of fibers [3].

Another design based on the formation of a laminated structure layer-by-layer reinforced with SiC fibers was suggested in the previous study, in which the novel preceramic paper-derived SiC_f_/SiC composites were successfully fabricated using spark plasma sintering (SPS) [1], which has the advantage of being faster than other processes—such as reactive melt infiltration (RMI) [7], chemical vapor infiltration (CVI), polymer infiltration pyrolysis (PIP) [8], etc. Preceramic paper, as a feedstock for sintering SiC, is a paper with a thickness of 0.5 mm that incorporates SiC powder with a size of about 4.5 μm, can be used as a raw for manufacturing ceramic substrates with complex geometric shapes. The detailed information on fabrication of SiC preceramic paper can be found in [9,10]. During SPS process, the organic components—such as hemicelluloses, cellulose, lignin, pulp fibers, etc.—decompose, and the remaining SiC particles is combined and densified with the SiC fibers to form the SiC_f_/SiC composites. The ‘nature’ of the layered-structure can also lead to the improvement of the mechanical properties such as flexural strength and fracture toughness of these composites [11]. It has been shown that the SiC_f_/SiC composites have obvious advantages as a structural material for nuclear reactors, such as high strength and quasi-ductility in comparison with SiC bulk materials, the improved mechanical properties are attributed to a combination of distinct toughening mechanisms—such as crack deflection, crack bridging, crack branching and delamination, pull-out, and layer rupture [1]. The main goal of this study was to analyze the microstructure of the paper-derived SiC_f_/SiC composites fabricated by SPS.

## 2. Materials and Methods

### 2.1. Fabrication of Paper-Derived SiC_f_/SiC Composites

The SiC_f_/SiC composites made of preceramic papers were fabricated using SPS. The manufacturing process of these specimens was presented in detail in [1]. The fabricated SiC_f_/SiC composites consist of the SiC layers derived from preceramic papers [9,10] and the layers of oxygen-free SiC-Nicalon™ fibers (Nippon Carbon Co., Tokyo, Japan). These fibers are slightly carbon-rich advanced fibers, which exhibit good stability under high levels of neutron irradiation [12]. The organic components in the preceramic paper decompose during sintering, and thus the porosity can be formed. In addition, during sintering a necking is formed between SiC particles, resulting in a uniform porous structure [1].

**Figure 1:**
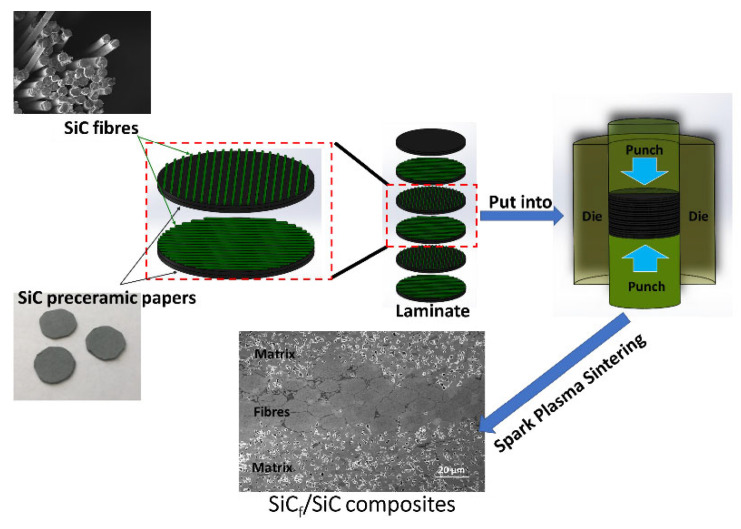
Schematic diagram of the fabrication process of the preceramic paper-derived SiC_f_/SiC composites.

A schematic representation of the manufacturing process of SiC_f_/SiC layered composites made from SiC filled preceramic papers and SiC fibers is shown in Figure 1. The uncoated SiC fiber bundles were placed between preceramic paper layers. The angle of 90° is set between two adjacent fiber layers to avoid poor mechanical properties across the fiber direction [13]. The fiber content was 10 wt %. In order to minimize the potential damage to SiC fibers at temperatures above 2300 °C [14], the samples in this work were sintered using SPS at 2100 °C and 2200 °C for 3 min and 10 min, respectively. The sintering pressure was increased to 100 MPa to reduce the porosity of the SiC_f_/SiC layered composites. The subsequent results confirm the effectiveness of this approach.

After the sintering, the cylindrical specimens with diameter of 20 mm were grinded, polished and rinsed with acetone and ethanol in an ultrasonic bath for 15 min.

### 2.2. Positron Annihilation Studies

The investigation of the defect structure was carried out by positron spectroscopy techniques. The positron lifetime spectroscopy (PLS) and Doppler broadening spectroscopy (DBS) were implemented. The PLS and DBS experiments were completed on the positron research platform (Institute of High Energy Physics of the Chinese Academy of Sciences, Peking). For the PLS, the ^22^Na isotope, transferred on polyimide film (DuPont™ Kapton^®^) was used as a positron source in this study. The source intensity was about 13 μCi. The maximum positron energy from the ^22^Na source is 1.275 MeV, which corresponds to the mean positron depth around 500 μm for SiC specimens. A pair of BaF_2_ scintillator detectors was used to detect the γ quanta released after positron generation and annihilation, and the positron lifetime spectrum was measured by fast–slow coincidence measurement technology. In the PLS experiment, the so-called “sandwich geometry” was used where the positron source was “sandwiched” between two identical samples under investigation [15]. To ensure that all emitted positrons are stopped and annihilated within the material, two identical samples were used in this study. For this reason, two identical samples were prepared by cutting one sample into two pieces.

The PLS had the time resolution of 195 ps. The cumulative count of each spectrum of positron lifetime was accumulated to the total of 2 × 10^6^ to ensure statistics. The electronic plug-in of the measurement system was the standard NIM from EG&G (USA). Decomposition of spectrums was carried out by multiple exponential components with the LT10 program (Poland). The contribution of the source was calculated by substituting $\tau_{\mathrm{source}}=0.382\;\mathrm{ns},I_{\mathrm{source}}=17.5\%$.

For the DBS, the ^22^Na isotope was used as the positron source, and the positron energy incident on the specimens was continuously adjustable in the range of 0–20 keV. The diameter of the positron beam was 5 mm.

The empirical formula for estimating the positron incident depth is

(1) $$R=\frac{40}{\rho }E^{1.6}$$

where *R* is the incident depth (nm), ρ is the material density (g/cm^3^), and *E* is the energy of the incident positron (keV) [16].

The Doppler broadening spectrum was collected by high-purity Ge detectors. The spectrums were accumulated to the total of 2 × 10^6^ counts in the Doppler-broadened annihilation peak. The energy resolution of spectrums was 1.2 at 511 keV. The annihilation properties are characterized by the S and W parameters. The total peak energy range of the collected gamma photon spectrum was 499.5–522.5 keV. The S parameter was defined as the ratio between the count in the energy range of 510.2–511.8 keV and the total peak (499.5–522.5 keV) count; the W parameter was defined as the ratio between counts in the energy range 513.6–516.9 keV and 505.1–508.4 keV to the total peak (499.5–522.5 keV) count.

### 2.3. Characterization by XRD, SEM, and Raman Spectroscopy

Phase composition of the composites were analyzed by X-ray diffraction (XRD) using D8 ADVANCE diffractometer (Bruker, USA). The scanning parameters: Cu-*K*_α_ radiation (λ = 0.154 nm), 2θ scan range 10–90°, accelerating voltage 20 kV, current 10 mA, scan speed 10°/min, sampling step 0.0143°. The phase composition was calculated by Rietveld method using JADE 6 software. The crystallite size was calculated using Scherrer equation [17]. Considering that both phases in SiC_f_/SiC composites are composed of SiC, it is difficult to obtain complete information—e.g., on the chemical composition—using only the scanning electron microscope (SEM). As a strong covalent compound, SiC has high Raman efficiency. Therefore, Raman scattering spectroscopy has been used as a powerful technical tool to characterize SiC-based materials [18].

In the present work, TESCA27N™ RISE microscope (Tschechische Republik, WITec GmbH, Germany) was used. Novel microscopy technique, RISE combines a confocal Raman microscope (CRM) with a SEM in an integrated microscope system [19]. For SEM analysis, the voltage of the electron gun is set to 5 keV. For the RAMAN, the scan step size is set to 50 μm. The wavelength of the laser is 532 nm. The power and diameter of the laser beam are 25 mw and <400 nm, respectively. The information on crystallite size in the specimens can be obtained via the linear relationship between the integral intensity ratio $I_{D}/I_{G}$ and $1/L_{a}$. For the laser wavelength used in this work (532 nm), the following empirical equation can be used to calculate the size of the crystal along the a-axis $L_{a}$ [20]

(2) $$L_{a}=4.4{\left[I_{D}/I_{G}\right]}^{-1}$$

## 3. Results and Discussion

**Figure 2:**
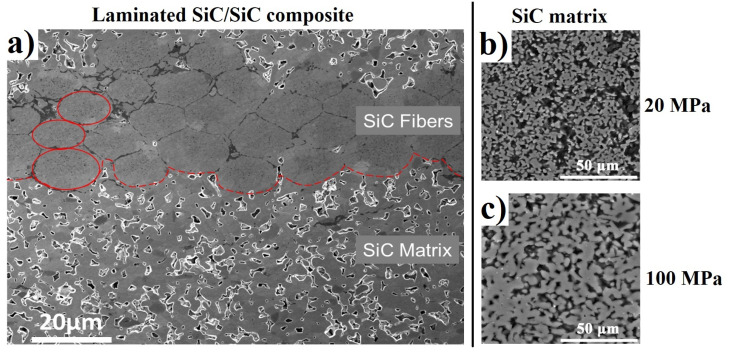
SEM image of SiC_f_/SiC composites sintered at 100 MPa (**a**), SiC matrix at 20 MPa (**b**), and 60 MPa (**c**).

Figure 2 shows the SEM images of the preceramic paper-derived SiC_f_/SiC composites. From the SEM image, the fiber-contained region (upper layer) and the matrix region (lower layer) are clearly observed. The morphology of the composites has a uniform irregular porous structure in the SiC layers formed by consolidation of the powder particles with the linear mean size of 4.5 μm, which is consistent with the size distribution of SiC particles in the as-received preceramic paper.

SEM analysis of SiC_f_/SiC composites shows that composites sintered at lower pressures (20–60 MPa) have a higher volume fraction of residual porosity when compared to materials sintered at pressures of 100 MPa [1].

### 3.1. Phase Composition

**Figure 3:**
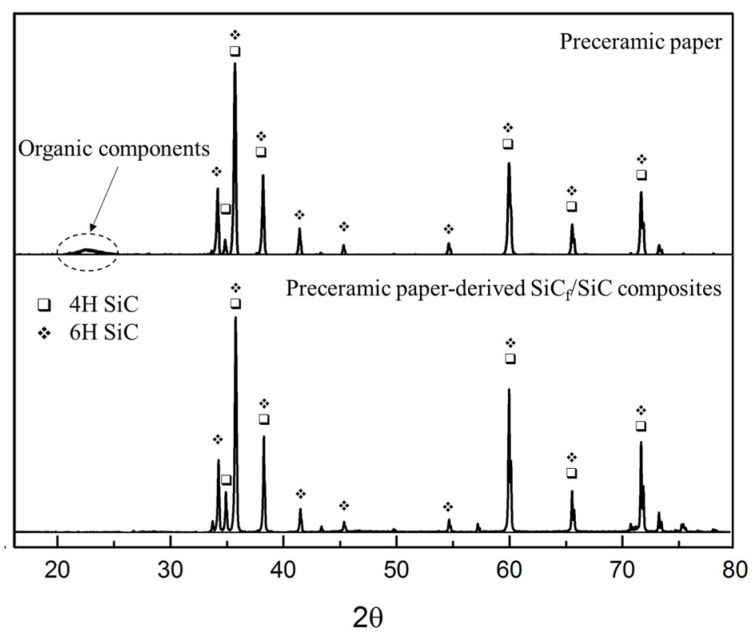
X-ray diffractograms of preceramic paper (**upper**), preceramic paper-derived SiC_f_/SiC composites (**lower**).

The spectrum is fitted by comparing with multiple PDF cards (Moissanite 4H, 29-1127; Moissanite 6H, 29-1131) using the JADE 6 program. As shown in Figure 3, no amorphous phase was observed in sintered preceramic paper-derived SiC_f_/SiC composites, that indicates complete decomposition of organic components in the preceramic papers during the sintering process. According to XRD, the SiC_f_/SiC composites consist of two crystalline polymorphic phases with hexagonal close package lattice (4H and 6H) lattice. The phase composition and crystallite size of the obtained composites are presented in Table 1.

Comparing the specimens obtained by sintering at 60 MPa and 100 MPa (at 2100 °C for 10 min), no significant changes in the phase composition were observed. At shorter sintering time (2100 degrees for 3 min), for the specimen obtained for 10 min under the same conditions, the proportion of 4H-SiC reduces from 28.1% to ~19%; correspondingly, the proportion of 6H-SiC phase increases from 71.9% to ~80%. In the specimen sintered at 2200 °C, the proportion of 4H-SiC phase decreases slightly, while the proportion of 6H-SiC increases correspondingly. Longer sintering time and higher sintering temperature also lead to higher degree of crystallization and larger crystallite size.

Moreover, 6H-SiC is very stable even when the temperature exceeds 2200 °C [21]. Therefore, a sintering temperature higher than 2100 °C leads to an increase in the proportion of 6H-SiC, and a longer sintering time allows more 4H-SiC to be converted into 6H-SiC.

### 3.2. Defect Structure

Table 2 shows the long-lived components of positrons for all specimens. The results show that the lifetime spectrum for each composite is characterized by the dominant short-lived component $\tau_{1}$ of 139 ps which corresponds to the bulk state of SiC calculated in [22]. For the composites sintered at 2100 °C, the dominant component $\tau_{1}$ has the intensity $I_{1}$ over 99%, and ~87% for that at 2200 °C.

Long-lived component $\tau_{3}$ (>2 ns) with extremely low intensity was observed in all specimens, this may be attributed to the pick-off annihilation of ortho-positronium (o-Ps) trapped on porous surfaces. The value of the $\tau_{3}$ decreases and its intensity $I_{3}$ increases in the specimen obtained at 2200 °C. This is mainly due to more very small pores inside the composite, which is consistent with the result of SEM analysis (Figure 2). In addition, a longer sintering time (10 min) also leads to a slightly increased $I_{3}$ value, but the influence is much lower than that of the temperature. Apart from this, no long-lived components were observed in the composites obtained at 2100 °C—i.e., there is no evidence that there are vacancy-type defects in these specimens.

Another long-lived component $\tau_{2}$ (=190 ps) only appeared in the composites obtained at 2200 °C, which is consistent with the theoretical value of silicon vacancy $V_{\mathrm{Si}}$ in 6H-SiC [23]. From XRD results, the transformation of 4H-SiC to 6H-SiC occurred at 2200 °C. Therefore, this transformation, accompanied by external loading, can cause distortions at the coherent interfaces leading to the formation of vacancy-type defects.

The formation energy of carbon vacancies $V_{C}$ (~20 eV) is much lower than that of silicon vacancies $V_{\mathrm{Si}}$ (~35 eV) [24]. However, no long-lived components corresponding to carbon vacancies of about 145 ps [23] were observed in any composites in this experiment. The binding energy of a positron to a carbon vacancy is less than 50 meV [22]. The strong Coulomb repulsion from the nearest neighboring Si atoms can result in weak localization of positrons at carbon vacancies. This may cause problems in detecting carbon vacancies by PLS. This explains why the lifetime component corresponding to the carbon vacancies is not detected in this experiment.

**Figure 4:**
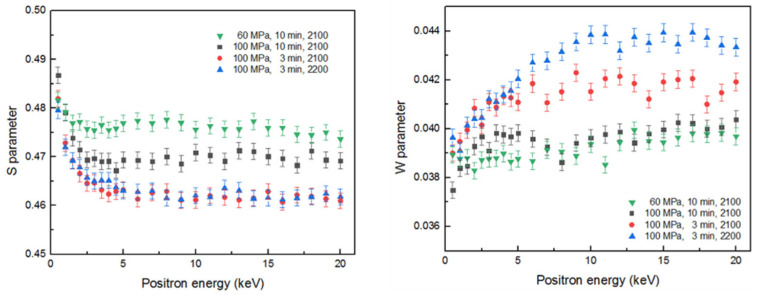
The dependence of line-shape S- and W-parameters of the DB spectra on the injection depth of positrons in specimens.

Figure 4 shows measured values of line-shape S- and W-parameters of the DB spectrum as a function of positron injection depth for specimens obtained under distinct conditions. The S-parameter is usually used for studying the size and concentration of defects such as vacancies, dislocations, and vacant clusters. W-parameters play the key role in the study of interstitial atoms and impurity atoms and precipitation phases [15].

The dependence of the S-parameter on the positron energy S(E) is represented by the superposition of S-parameters at the surface and those in the specimen interior

(3) $$S\left(E\right)=F_{s}\left(E\right)S_{s}+\left[1-F_{s}\left(E\right)\right]S_{i}$$

where $S_{s}$ is the characteristic S-parameters at the surface of the specimen;$\;S_{i}$ is the S-parameters in the specimen interior; $F_{s}\left(E\right)$ is the annihilations ratio of injected positrons annihilating at the surface. When the positron injection energy is low (the corresponding depth range is 0–160 nm or 0–5 keV), the S-parameters of almost all specimens show rapid decline with the injection energy increase. This is mainly due to part of positron diffusing back to the near surface and the formation of the ortho-positronium (o-Ps), causing the S parameter to decrease. In addition, the near-surface region is often complex in structure, and the positrons diffused to the surface annihilate in the surface state, usually with large S parameters [25]. As the injected energy continues to rise, the S-parameters are independent of injected positron energy in the range above ~10 keV.

It can be seen from the S–E curves that the composite material obtained by sintering for 3 min has lower S parameter than those of the composites sintered for 10 min. This is explained by the fact that a longer duration at high temperature leads to a higher degree of phase transformation and the formation of defects [26]. Furthermore, higher sintering pressure (100 MPa) also results in lower S-parameter. It is believed that the difference in S-parameters is mainly due to the difference in porosity and grain size, since the isolated silicon vacancies already disappear below 1000 °C [22], which is far below the sintering temperature. This also corresponds to the results of the positron lifetime spectrum for specimens—i.e., no long-lived components are found in the various specimens.

The specimen prepared at 60 MPa has the highest S-parameter, and compared with other specimens sintered at the pressure of 100 MPa, its S-parameter has reached the constant at lower energy of positron. Since the presence of more pores makes the positron diffusion length much shorter (about a few nm), the probability of positron diffusion back to the surface is lower, which in turn reduces the influence of surface effects on the S-parameters. The value of the S parameter is considered to be determined by the pore size. The S parameter is larger when the pore size is larger [27].

**Figure 5:**
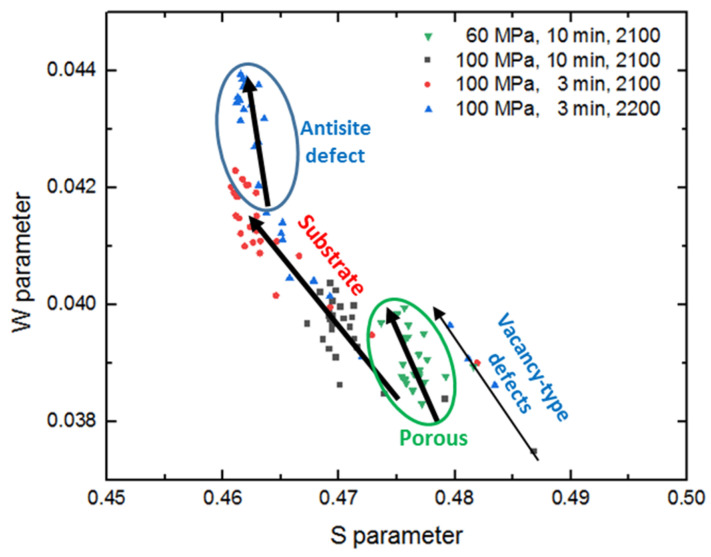
Dependence of line-shape S-parameters of the DB spectra on W-parameters.

Measured values of line-shape S-parameters of the DB spectrum as the function of W-parameters for specimens obtained under distinct conditions are presented in Figure 5.

From the W–S curve, the annihilation traces of positrons in the samples can be seen. It was observed that positrons are annihilated in the substrates for most specimens. However, for the specimen prepared at 60 MPa, the positrons are mainly annihilated in the pores due to the larger size of the pores (green circle). Compared to the specimen fabricated in 10 min at 100 MPa, the pore size is smaller due to the higher sintering pressure, and the W–S curve is closer to the substrate area.

For the specimen sintered at 2200 °C, the pores are mainly reflected in the nanometer level, the S parameter does not increase significantly compared to the matrix. Thus, compared to the specimen fabricated at 2100 °C (using the same other sintering conditions), no difference in S-parameters can be seen. Considering that the value of the W-parameter of the specimen fabricated at 2200 °C is higher than that prepared at 2100 °C, it can be concluded that the antisite defect was generated by the C atoms filling the Si vacancies during the sintering process, which increased probability of the annihilation of the positron with the high momentum electrons. This can also be confirmed by comparing the ratio curve [28] or the energy spectra of carbon [29] and SiC [30]—i.e., in the energy spectrum graphite crystals have larger proportion of the integrated area of the region corresponding to high-momentum electrons. The positron lifetime does not change significantly when the silicon vacancy transforms into the antisite defects [27], thus the components of the antisite defects in the result of positron lifetime are not distinguished from that of the Si vacancy. This conclusion is consistent with the conclusions of the PLS and the XRD analysis from the previous section—i.e., phase transition rate of SiC is accelerated at 2200 °C—the pressure of sintering causes distortion at the coherent interface, resulting in defects. The antisite defect is reflected in the blue circular area on the W–S curve. It was observed that the positrons in the specimen fabricated at 2200 °C are annihilated in the substrate and the antisite defect respectively.

**Figure 6:**
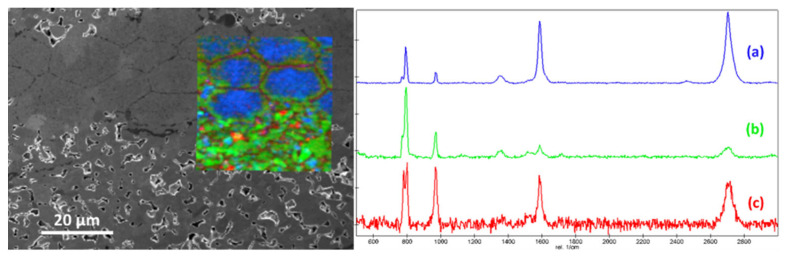
Position distribution of the Raman spectrum of the SiC_f_/SiC specimen: left RISE image; right Raman spectrum: where (**a**) is fiber, (**b**) is matrix, and (**c**) is porosity.

Raman scattering spectroscopy can be used to obtain information on the microstructure of materials using different spectral characteristics, such as position of peak, intensity of peak, full width at half maximum (FWHM) of peak, etc. [31]. Furthermore, with the exception of 2H and 3C-SiC, all SiC polytypes are constructed of a mixture of cubic and hexagonal stacking of SiC double layers, from which specific Raman spectra can be derived [32], information on the presence of different polytypes in the SiC specimens can be obtained [18]. For the scan data collected by RISE, the obtained Raman scattering spectra are matched with the positions of the SEM images to obtain the position distribution of the Raman spectrum. The RISE image is extracted in Figure 6.

Information on the stoichiometry of materials can be obtained by means of Raman spectroscopy, the carbon-rich characteristics of SiC fibers become the key to distinguishing between the fiber and the matrix, and the characterization of the carbon is one of the most advantageous capabilities of Raman spectroscopy. As demonstrated in Figure 6, the color of the Raman spectrums line is the same as the color of the corresponding areas in the RISE image. The image can clearly distinguish the SiC fibers (blue) and the SiC layer (green) in the specimen. The few red areas in the matrix represent the carbon remaining after the decomposition of the organics in the preceramic paper during sintering. It can be seen from the RISE image that this free carbon is in the pores of the SiC layer.

From spectrum (a), it is observed that the spectrum in the region from 200 ${\mathrm{cm}}^{-1}$ to 2800 ${\mathrm{cm}}^{-1}$ has several manifested well-defined Raman bands. SiC in the fibers gives rise to the band at ~830 ${\mathrm{cm}}^{-1}$ and ~930 ${\mathrm{cm}}^{-1}$ [33], while the spectrum shows the main Raman features of graphite type [34]: D band ~1350 ${\mathrm{cm}}^{-1}$, G band ~1582 ${\mathrm{cm}}^{-1}$, G* band ~2450 ${\mathrm{cm}}^{-1}$, and G’ band ~2680 ${\mathrm{cm}}^{-1}$.

The observed obvious Raman characteristic peaks for carbon are caused by the Si/C stoichiometric ratio in the fibers being less than 1:1, and the excess C forming a C–C bond structure. Therefore, these peaks reflect the presence of the carbon packets in the fibers [35]. Considering that the LO and TO vibration dipole moments of the C–C bond do not change, the Raman scattering efficiency of the C–C bond is an order of magnitude higher than that of the Si–C bond [36]. Even if the share of the C–C bond structure is less than that of the Si–C bond, the Raman scattering peaks of C–C bond can be observed clearly. The spectrum shows the obvious G band and the very strong G’ band, which are prominent features in the Raman spectra of monolayer graphene or turbostratic graphite [34].

**Figure 7:**
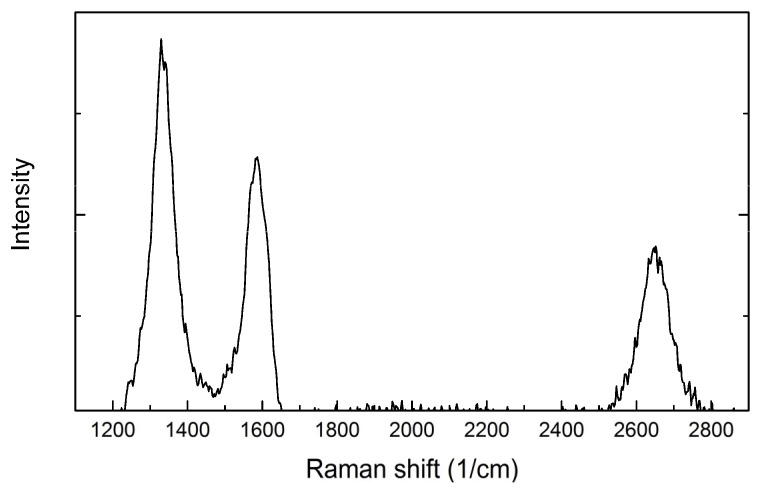
Raman spectrum of carbon in unsintered SiC fiber.

Comparing the Raman spectrum of the SiC fiber before sintering (Figure 7), it can be observed that the C in the initial SiC fiber is mainly in the form of amorphous carbon [37], rather than the turbostratic graphite (or graphene) in the SiC fiber of the sintered SiC_f_/SiC composites. The turbostratic graphite (or graphene) may be caused by the diffusion of carbon from the preceramic paper to the fiber surface during the sintering process. In a high-temperature environment, the Si atoms from SiC sublime and break away, the remaining C atoms including the penetrated C atoms are combined, thereby forming graphite on the surface of SiC [38]. The results of Malard et al. showed that graphite heat-treated at ~2200 °C exhibits a typical spectrum of a turbostratic graphite, which is composed of only one Lorentzian component [34], which is consistent with the G’ peak in Figure 6. Considering that the sintering temperature of the material is 2100–2200 degrees, it is inferred that the observed G’ may with a high probability be the typical peak of turbostratic graphite.

The average crystal size along the a-axis direction of the graphite in the fiber of the SiC_f_/SiC composites was calculated according to the above formula is ~41 nm.

**Figure 8:**
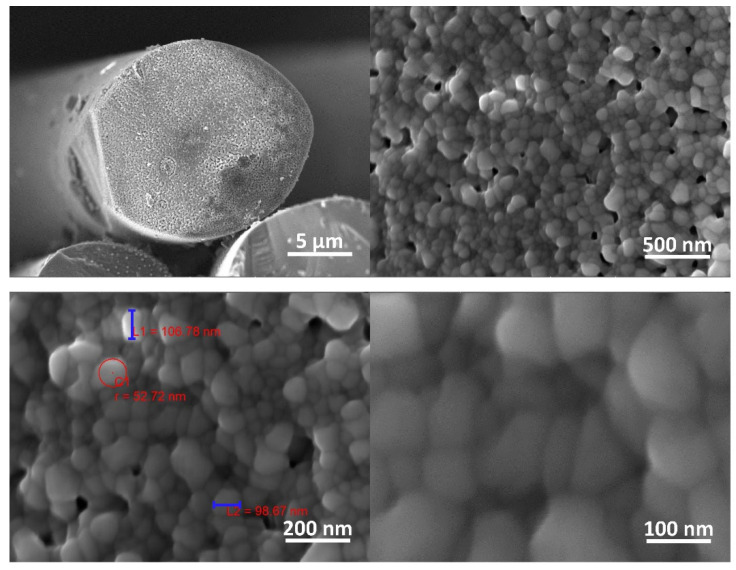
SEM-images of the SiC fiber under various magnifications.

Figure 8 shows the SEM images of the SiC fiber under various magnifications. The SiC particles can be observed in the SEM images, whose measured size does not exceed ~100 nm. The pores between the SiC particles provide channels for carbon diffusion during the sintering process, forming turbostratic graphite in an oxygen-free, high-temperature environment.

**Figure 9:**
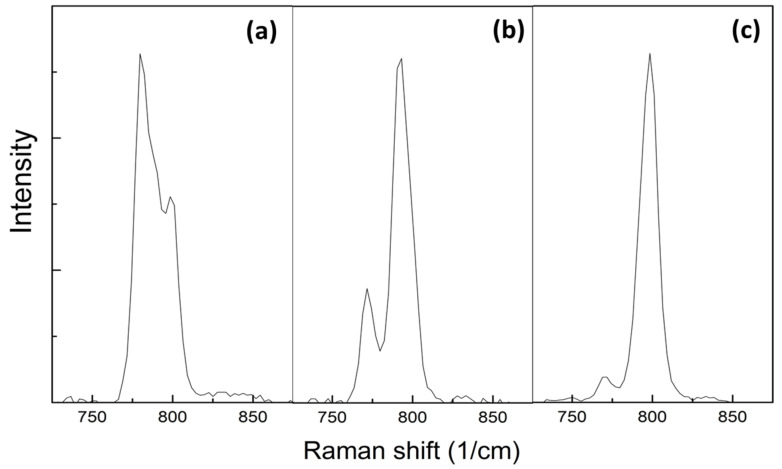
Details of the TO peaks for the SiC matrix of the novel preceramic paper-derived SiC_f_/SiC Composites by sintering at 2100 °C for 3 min (**a**); matrix with other sintering conditions (**b**); and SiC fiber (**c**).

Table 3 shows the TO peak positions of SiC in each specimen. Figure 9 shows the detailed profiles of the TO peaks for the SiC layer (a,b) and SiC fiber (c) of the composites. The TO peak for the SiC layer is obviously split, and the overall profile is closer to 6H-SiC, while the detail for composite sintered at 2100 °C for 3 min is clearly observed the 4H-SiC [39], which is consistent with the results of XRD.

For the 3C–SiC, there are two peaks, one at 796 cm^−1^ that is assigned to the transverse optical (TO) phonon peak (double degeneration) and the other at 972 ${\mathrm{cm}}^{-1}$ to the longitudinal optical (LO) phonon peak [32]; for 4H-SiC, there are TO peaks at 775 and 795 cm^−1^ and LO at 967 ${\mathrm{cm}}^{-1}$ [39]; and for the 6H–SiC, there are TO peaks at 767 and 788 cm^−1^ and LO at 967 ${\mathrm{cm}}^{-1}$ [21,39]. According to the spectrum for the fiber, it can be determined that the fibers in SiC_f_/SiC composites are mainly composed of 6H-SiC. The positions of the TO peak for 4H-, and 6H-SiC are 794, 796, and 788 cm^−1^, respectively. Except for the composite sintered at 2100 °C for 3 min, the TO peak position for the matrix in the other specimens is at 792 cm^−1^, which is significantly red shifted compared with that for the composite fabricated at 2100 °C for 3 min (~799 cm^−1^). This red shift is due to longer sintering time or the sintering temperature of 2200 °C, which cause more phase transition to 6H-SiC. This is consistent with the XRD and PS data. Additionally, the peak position of each specimen is slightly blue-shifted compared with the standard value, which is caused by compressive stress introduced by the sintering process.

## 4. Conclusions

We have evaluated the microstructure of the novel preceramic paper-derived SiC_f_/SiC composites fabricated by the spark plasma sintering method with different sintering conditions. The influence of the sintering pressure and time on defect structure of the composites was revealed.

Based on the obtained results, the following conclusions were made:

1. Different sintering conditions change the phase composition of the fabricated composites. Compared to a 2100 °C, higher sintering temperature (2200 °C) accelerates the phase transition in the material to 6H-SiC; compared to 3 min, a longer sintering time (10 min) results in an increase in the proportion of 6H-SiC. Nanopores are formed in the specimen sintered at 2200 °C.
2. The sintering process removes vacancy-type defects in the material. However, higher sintering temperature (2200 °C) leads to excessively high phase transition rate, further introducing vacancy-type defects.
3. A phase is observed in the fibers of SiC_f_/SiC composites that is believed to be turbostratic graphite. The turbostratic graphite (or graphene) may be caused by the diffusion of carbon from the residual cellulose fibers surface during the sintering process.

## Figures and Tables

**Table 1:** Results of X-ray diffraction analysis of the preceramic paper-derived SiC_f_/SiC composites.

Specimen	Sintering Pressure (MPa)	Sintering Time (min)	Sintering Temperature (°C)	Phase Ratio (wt %)		Crystallite Size (nm)
				4H	6H	
1	60	10	2100	19.5	80.5	181.3
2	100	10	2100	18.4	81.6	194.2
3	100	3	2100	28.1	71.9	112.5
4	100	3	2200	27.4	72.6	156.1

**Table 2:** Measured positron lifetime in the preceramic paper-derived SiC_f_/SiC composites.

Specimen	Sintering Pressure (MPa)	Sintering Time (min)	Sintering Temperature (°C)	$\tau_{1}\;(\mathrm{ps})$	$I_{1}\;(\%)$	$\tau_{2}\;(\mathrm{ps})$	$I_{2}\;(\%)$	$\tau_{3}\;(\mathrm{ns})$	$I_{3}\;(\%)$	$\bar{\tau }\;(\mathrm{ps})$
1	60	10	2100	139	99.5	-	-	2.8	0.5	149
2	100	10	2100	139	99.5	-	-	2.6	0.5	149
3	100	3	2100	139	99.6	-	-	2.7	0.4	148
4	100	3	2200	139	87.2	190	11.7	2.1	1.1	164

**Table 3:** Peak position of the TO vibration mode of the preceramic paper-derived SiC_f_/SiC composites.

Specimen	Sintering Pressure (MPa)	Sintering Time (min)	Sintering Temperature (°C)	$\mathrm{Peak}\;\mathrm{Position}\;\mathrm{of}\;\mathrm{TO}\;\mathrm{for}\;\mathrm{SiC}\;\mathrm{Fiber}\;(cm^{-1})$	$\mathrm{Peak}\;\mathrm{Position}\;\mathrm{of}\;\mathrm{TO}\;\mathrm{for}\;\mathrm{SiC}\;\mathrm{Matrix}\;(cm^{-1})$
1	60	10	2100	770	772
				798	793
2	100	10	2100	770	772
				798	792
3	100	3	2100	770	779
				798	799
4	100	3	2200	770	772
				798	793

## Data Availability

The data presented in this study are available on request from the corresponding author.
